# Supplementary material for: Amplicon Sequencing Reveals Novel Fungal Species Responsible for a Controversial Tea Disease
Source: J Fungi (Basel). 2022 Jul 26;8(8):782. doi: 10.3390/jof8080782 (PMC9394346; doi:10.3390/jof8080782)
Supplement: Supplementary file 1 [file jof-08-00782-s001.zip › jof-1758693-supplementary.pdf]

Amplicon Sequencing Reveals Novel Fungal Species Responsible for a Controversial Tea Disease

Yunqiang He [1,2,3,4], Yan Li [1,5], Yulin Song [5], Xingming Hu [6], Jinbo Liang [7], Karim Shafik [2,3,4,8], Dejiang Ni [1,2] and Wenxing Xu [1,2,3,4,*]

1 Hubei Hongshan Laboratory, Wuhan 430070, China; heyunqiang@webmail.hzau.edu.cn (Y.H.); ly632973748@163.com (Y.L.); nidj@mail.hzau.edu.cn (D.N.)
2 Key Laboratory of Horticultural Plant Biology (Ministry of Education), Wuhan 430070, China; karim.awad@alexu.edu.eg
3 College of Plant Science and Technology, Huazhong Agricultural University, Wuhan 430070, China
4 Key Lab of Plant Pathology of Hubei Province, Wuhan 430070, China
5 Tea Industry Office, Agriculture and Rural Bureau of Zigui County, Yichang 443699, China; songyulin222@163.com
6 Agriculture and Rural Bureau of Enshi, Enshi 445015, China; xiangjun@tricaas.com
7 Academy of Agricultural Sciences of Enshi, Enshi 445002, China; esljb2022@163.com
8 Department of Plant Pathology, Faculty of Agriculture, Alexandria University, Alexandria 21526, Egypt
* Correspondence: xuwenxing@mail.hzau.edu.cn

Supplementary tables and figures:

Two tables and three figures

Table S1. Source of isolates and GenBank accession number in this study

Species	Strain number	Status	Original substrate	Country	GenBank accession numbers			
					LSU	ITS	RPB2	TUB
D. aquatica	CGMCC 3.18349; LC 5556	T	*Water*	China	KY742209	KY742055	KY742140	KY742297
	LC 5555		*Water*	China	KY742210	KY742056	KY742141	KY742298
D. bellidis	CBS 714.85; PD 74/265	R	*Bellis perennis*	Netherlands	GU238046	GU237904	KP330417	GU237586
	PD 94/886		*Bellis* sp.	Netherlands	GU238047	GU237923	N/A	GU237587
D. brunneospora	CBS 115.58; DSM 62044	T	*Tanacetum coccineum*	Germany	KT389723	KT389505	KT389625	KT389802
D. cari	CPC33114		*Coriandrum sativum*	Canada	MH327863	MH327827	N/A	MH327901
	CPC33113		*Coriandrum sativum*	Canada	MH327862	MH327826	N/A	MH327900
D. chenopodii	CBS 129.93		*Chenopodium quinoa*	Peru	GU238056	GU237776	MT018172	GU237592
D. chloroguttulata	CGMCC 3.18351; LC 7435	T	*Air*	China	KY742211	KY742057	KY742142	KY742299
	LC 8122		*Air*	China	KY742212	KY742058	KY742143	KY742300
D. ellipsoidea	CGMCC 3.18350; LC 7434	T	*Air*	China	KY742214	KY742060	KY742145	KY742302
	LC 8123		*Air*	China	KY742215	KY742061	KY742146	KY742303
D. infuscatispora	CGMCC 3.18356; LC 8128	T	*Chrysanthemum indicum*	China	KY742221	KY742067	KY742152	KY742309
	LC 8129		*Chrysanthemum indicum*	China	KY742222	KY742068	N/A	KY742310
D. kooimaniorum	CBS 144951; JW 27006	T	*garden soil*	Netherlands	MN823299	MN823448	MN824474	MN824622
D. macrophylla	CGMCC 3.18357; LC 8131	T	*Hydrangea macrophylla*	Italy	KY742224	KY742070	KY742154	KY742312
	LC 8132		*Hydrangea macrophylla*	Italy	KY742225	KY742071	KY742155	KY742313
D. macrostoma	CBS 482.95		*Larix decidua*	Germany	GU238099	GU237869	KT389609	GU237626
	CBS 529.66; PD 66/521	R	*Malus sylvestris*	Netherlands	GU238098	GU237885	N/A	GU237625
D. microchlamydospora	CBS 105.95	T	*Eucalyptus* sp.	UK	GU238104	FJ427028	KP330424	FJ427138
D. molleriana	CBS 229.79; LEV 7660	R	*Digitalis purpurea*	New Zealand	GU238067	GU237802	KP330418	GU237605
	CBS 109179; PD 90/835–1		*Digitalis* sp.	Netherlands	GU238066	GU237744	N/A	GU237604
D. negriana	CBS 358.71	R	*Vitis vinifera*	Germany	GU238116	GU237838	KT389610	GU237635
	ICMP 10845; LC 5249		*Vitis vinifera*	Yugoslavia	KY742227	KY742073	N/A	KY742315

D. ocimicola	CGMCC 3.18358; LC 8137	T	*Ocimum* sp.	China	KY742232	KY742078	N/A	KY742320
	LC 8138		*Ocimum* sp.	China	KY742233	KY742079	N/A	KY742321
D. pteridis	CBS 379.96	T	*Pteris* sp.	Netherlands	KT389722	KT389504	KT389624	KT389801
D. rhei	CBS 109177; LEV 15165; PD 2000/9941	R	*Rheum rhaponticum*	New Zealand	GU238139	GU237743	KP330428	GU237653
	BRIP 5562; LC 5251		*Rheum rhaponticum*	Australia	KY742237	KY742083	KY742163	KY742325
D. rosea	BRIP 50788	T	plant tissue	Australia	KT287003	KT338640	N/A	KT286945
D. segeticola	CGMCC 3.17489; LC1636	T	*Cirsium segetum*	China	KP330455	KP330443	KP330414	KP330399
	CGMCC 3.17498; LC1635		*Cirsium segetum*	China	KP330454	KP330442	KP330413	KP330398
D. senecionicola	CBS 160.78; LEV 11451	R	*Senecio jacobaea*	New Zealand	GU238143	GU237787	N/A	GU237657
D. subrosea	CBS 733.79	T	*Abies alba*	France	MN943747	MN973540	MT018174	MT005643
D. suiyangensis	CGMCC 3.18352; LC 7439	T	*Air*	China	KY742243	KY742089	KY742168	KY742331
	LC 8144		*Air*	China	KY742244	KY742090	KY742169	KY742332
D. tanaceti	BRIP 50785		plant tissue	Australia	KT287022	KT338641	N/A	KT286974
D. theifolia	CGMCC 3.20887; JYC-1-9	T	*Camellia sinensis*	China	ON390998	OM203465	ON391008	ON391012
	JYC-1-6		*Camellia sinensis*	China	ON390997	ON392757	ON391007	ON391011
	SZX-1-9		*Camellia sinensis*	China	ON390999	ON392755	ON391009	ON391013
	SZX-1-10		*Camellia sinensis*	China	ON391000	ON392756	ON391010	ON391014
D. theae	CGMCC 3.20886; WJT-2-3	T	*Camellia sinensis*	China	ON390994	OM203464	ON391004	ON391017
	SZX-3-2		*Camellia sinensis*	China	ON390991	ON378798	ON391001	ON391015
	SZX-3-7		*Camellia sinensis*	China	ON390992	ON378797	ON391002	ON391020
	WJT-1-2		*Camellia sinensis*	China	ON390993	ON378795	ON391003	ON391016
	WJT-2-7		*Camellia sinensis*	China	ON390995	ON378796	ON391005	ON391018
	WJT-2-9		*Camellia sinensis*	China	ON390996	ON378799	ON391006	ON391019
D. variabilis	CBS 2554.79	T	*Vitis vinifera*	Italy	MN943751	MN973544	MT018182	MT005647
D. viburnicola	CBS 523.73; PD 69/800	R	*Viburnum cassioides*	Netherlands	GU238155	GU237879	KP330430	GU237667
Neoascochyta paspali	CBS 560.81; PD 92/1569	T	*Paspalum dilatatum*	New Zealand	GU238124	FJ427048	KP330426	FJ427158

T: ex-type strain; R: representative strin.

Table S2 Data preprocessing statistics and quality control for SZX, JYC, WJT, and NC samples

No.	Raw PE	Raw Tags	Clean Tags	Effective Tags	Base (nt)	Avg Len (nt)	Q20	Q30	GC (%)	Effective (%)
SZX	89,258	86,314	86,167	66,187	15,504,409	234	99.44	98.04	51.26	59.41
JYC	99,175	86,653	82,771	63,279	15,152,198	239	97.81	94.53	51.19	63.81
WJT	106,036	64,950	64,575	62,994	14,742,906	234	99.19	97.37	48.25	74.15
NC	85,060	82,588	82,297	64,043	15,521,349	242	99.37	97.75	48.05	75.29

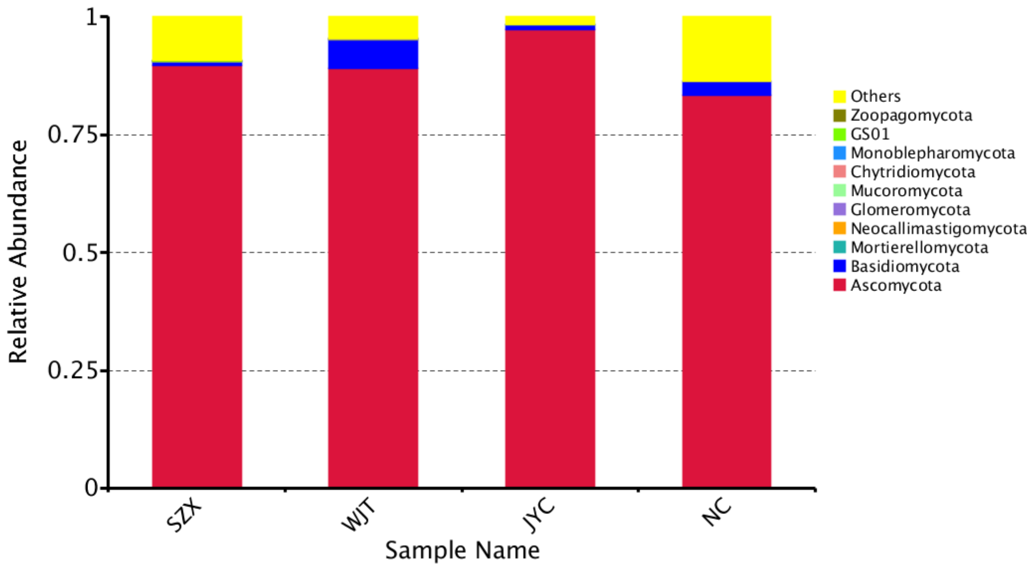

Figure S1. Bar graph representing the relative abundance of the fungal phyla revealed by amplicon sequencing in samples SZX, WJT, JYC, and NC. Only ten fungal species with leading relative abundance in each sample were selected and involved in the analysis. Others indicate to the sum of the relative abundances of all the phyla except these ten mentioned fungi.

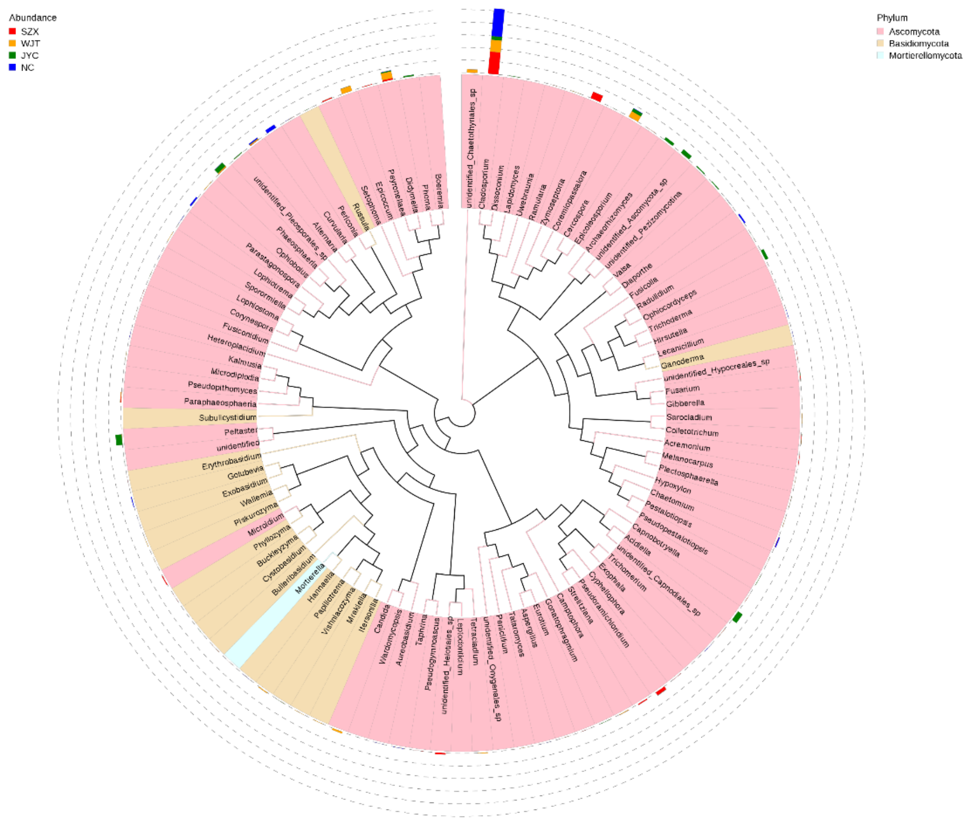

Figure S2. Phylogenetic relationship of the fungal genera revealed by amplicon sequencing in samples SZX, WJT, JYC, and NC. Only 100 fungal species with leading relative abundance in each sample were selected and involved in the analysis. The color of the branch indicates its corresponding phylum, and each color represents a phylum. The bars beside the genera refer to the abundance corresponding to the bar height, and the different colors indicate the different samples, i.e., red, brown, green and blue for SZX, WJT, JYC, and NC, respectively.

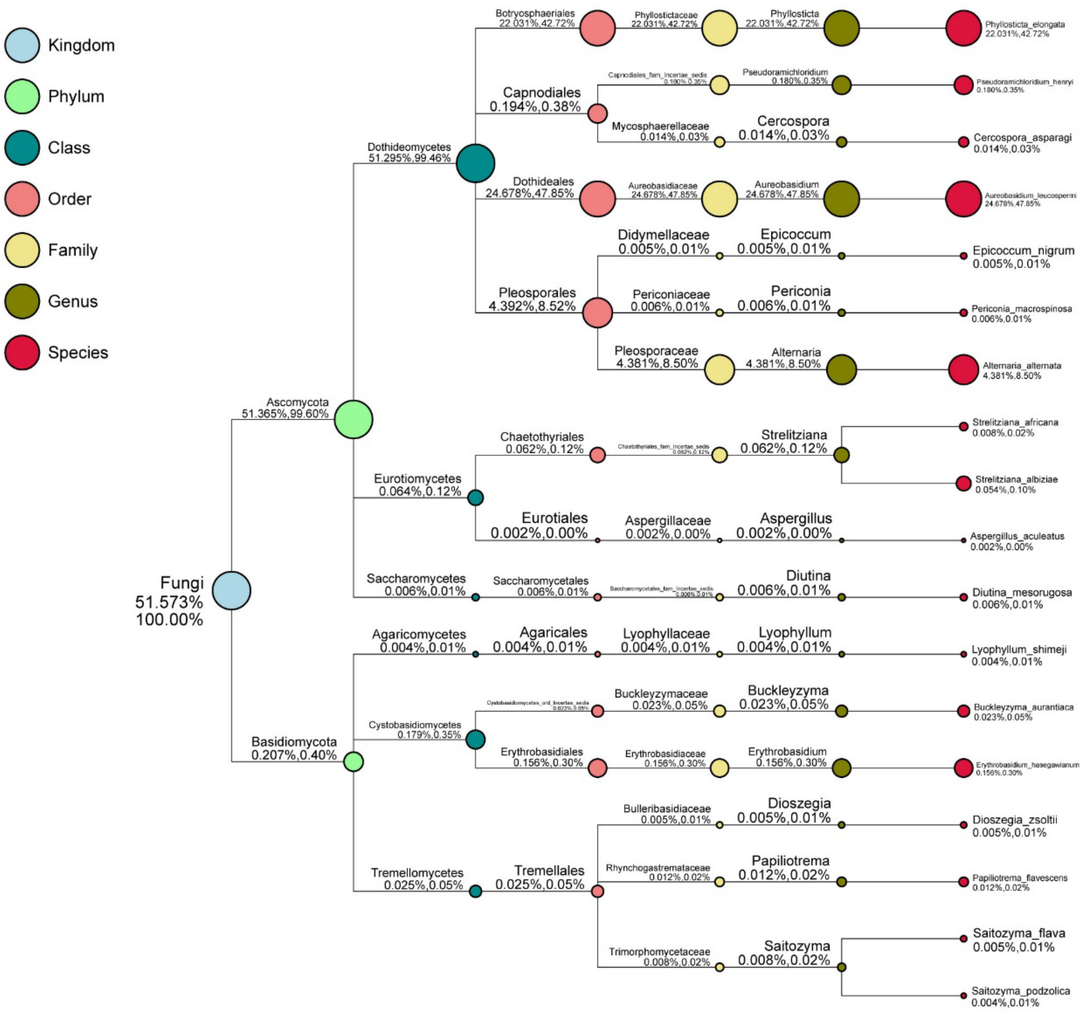

Figure S3. Phylogenetic tree of fungi revealed by amplicon sequencing in sample WH at diverse taxonomic situation. Only 20 fungal species with leading relative abundance were selected from each sample and involved in the analysis. The circles with different colors on the upper-left position refer to the diverse samples with the names beside them; the circles in different nodes indicate different taxonomic levels, with the different colors and sizes referring to the different sample composition and their relative abundance, respectively. The two numbers below the category name represent the proportion of their relative abundance accounting for all the fungal categories and the selected ones for the former and later, respectively.
